# Granulated straw incorporation with rotary tillage increases the content of soil organic carbon fractions and available nutrients and shifts bacterial communities in East China

**DOI:** 10.3389/fpls.2025.1520760

**Published:** 2025-07-31

**Authors:** Jianxin Dong, Ping Wang, Ping Cong, Wenjing Song, Xuebo Zheng, Na Liu, Yi Wang, Xin Xiao, Zhen Zhai, Yuyi Li, Huancheng Pang

**Affiliations:** ^1^ Key Laboratory of Tobacco Biology and Processing, Ministry of Agriculture and Rural Affairs, Tobacco Research Institute of Chinese Academy of Agricultural Sciences, Qingdao, China; ^2^ College of Tourism, Xinyang Normal University, Xinyang, China; ^3^ Zhucheng Experimental Station, Zhucheng Branch, Weifang Tobacco Co., Ltd., Weifang, China; ^4^ Key Laboratory of Ecological Environment and Tobacco Quality, Zhengzhou Tobacco Research Institute of China National Tobacco Corporation, Zhengzhou, China; ^5^ Institute of Agricultural Resources and Regional Planning, Chinese Academy of Agricultural Sciences (CAAS), Beijing, China

**Keywords:** granulated straw incorporation, tillage method, soil organic carbon, soil available nutrients, bacterial community

## Abstract

**Introduction:**

Granulated straw incorporation is a novel approach designed to enhance straw decomposition and improve soil fertility. However, the effects of different straw incorporation amounts under deep tillage and rotary tillage on soil available nutrients, soil organic carbon (SOC) fractions, bacterial communities, and crop yield remain unclear.

**Methods:**

In a 3-year field experiment, three granulated maize straw amounts (G1, 2,250; G2, 4,500; and G3, 6,750 kg hm^−2^) and two tillage methods (T, deep tillage; and R, rotary tillage) were applied to evaluate their impacts on SOC fractions, available nutrients, bacterial communities, and flue-cured tobacco yield.

**Results and Discussion:**

Compared with conventional tillage (RG0), granulated straw incorporation significantly increased SOC content. Over the 3 years, the SOC content in the 0–20 and 20–40 cm soil layers increased by 4.40%–23.46% and 5.36%–39.21% (*p* < 0.05), respectively. Moreover, the incorporation of higher straw amounts significantly increased the content of dissolved organic carbon (DOC) and microbial biomass carbon (MBC). Specifically, the RG3 treatment significantly increased DOC content in both soil layers in 2016 and 2017, while TG3 showed the greatest increase in 2018. In addition, RG2 and RG3 consistently enhanced MBC content across both layers throughout the 3 years. During the tobacco growing period, soil ammonium nitrogen (NH_4_^+^–N), nitrate nitrogen (NO_3_^−^–N), and available potassium (AK) contents increased with higher straw amounts in both soil layers. The RG2 treatment notably enhanced the bacterial α diversity and increased the relative abundance of phyla Firmicutes and Gemmatimonadota in the 20–40-cm layer. Network analysis identified AK as a key nutrient influencing bacterial community structure under both tillage methods. Structural equation modeling further revealed that SOC fractions were primarily regulated by nutrient factors under rotary tillage, while under deep tillage, bacterial richness and AK played dominant roles. To improve soil quality and crop productivity, the incorporation of a medium amount of granulated straw combined with rotary tillage is recommended as a sustainable practice for flue-cured tobacco cultivation.

## 1 Introduction

Over 3 billion tons of straw is produced globally each year (Wang et al., 2021). China, as a major agricultural country, generates over one-third of the world’s straw. However, in the eastern and southern provinces of China, more than 30% of this straw is either left in the fields or burned outdoors, leading to considerable resource waste and environmental pollution (Wang et al., 2018). After the Chinese government issued the ban on straw burning (State Council of China, 2014), the efficient utilization of straw has become a key strategy for managing the large quantities generated annually.

Granulated straw returning is a novel straw management method in which straw is crushed, extruded, and granulated before being returned to the field. This approach not only accelerates soil fertility enhancement but also offers a practical solution for large-scale straw due to its higher decomposition rate (31.68% greater than that of conventional crushed straw) and higher bulk density (nearly five times that of conventional crushed straw) (Wang et al., 2017a). It has thus been regarded as a win–win strategy for resource recycling and environmental protection. Granulated straw returning with different tillage methods creates a distinct distribution of soil nutrients and organic carbon pool by affecting the soil physicochemical and biological characteristics (Zhang et al., 2017). Rotary tillage has been shown to rapidly increase soil available nutrients and soil organic carbon (SOC) in the 0–15-cm soil layer– (Zhang L. et al., 2018), while deep tillage is more effective for long-term carbon sequestration in the 20–40- and 40–60-cm soil layers (Liu N. et al., 2021). In addition, the amount of incorporated straw is also a critical factor influencing changes in soil nutrients, SOC, and microbial communities. It is widely recognized that increasing straw incorporation can enhance soil available nutrients and SOC content due to the high concentrations of C, N, P, and K present in straw (Cong et al., 2020). The compact volume of granulated straw allows for greater application amounts compared to conventional straw. However, the appropriate amount of granulated straw to incorporate under rotary tillage and deep tillage for enhancing soil available nutrients and SOC content remains inadequately studied.

Soil microorganisms play a crucial role in straw decomposition and nutrient cycling (Latifmanesh et al., 2020) and are widely regarded as sensitive biological indicators of soil fertility (Lehmann et al., 2020; Latifmanesh et al., 2020). Straw incorporation has been shown to positively impact microbial activity and enhance microbial diversity (Zhang X. F. et al., 2018). As a rich carbon source, decomposing straw stimulates SOC turnover and promotes the abundance and diversity of carbon-related microorganisms (Cong et al., 2020). However, the role of tillage in regulating soil microbial communities remains controversial. Some studies have highlighted that tillage methods with minimal soil disturbance are more conducive to increasing the abundance of specific soil microbial functional groups, promoting greater soil microbial diversity, and enhancing the metabolic activity of soil microbial communities (Mbuthia et al., 2015; Wang et al., 2017c). Nevertheless, the benefits of deep tillage for improving soil microbial biomass and activity have also been confirmed (Li et al., 2019). The proportions of soil solid, liquid, and gas phases adjusted by deep tillage provided a more suitable environment for microbes (Liu et al., 2022). Tillage methods can also change the distribution of straw in the soil, thereby indirectly impacting soil microbial communities. Liu et al. (2022) indicated that straw incorporation with deep tillage has significant potential for enhancing microbial community characteristics in both subsoil and topsoil. Furthermore, in short-term experiments, the soil bacterial community is widely considered a reliable bioindicator for assessing changes in the soil environment owing to its highly sensitive response to environmental shifts. Based on existing theories, this study focuses on analyzing how the bacterial community of granulated straw may potentially respond differently under two different tillage methods and their interactions with soil available nutrients and SOC fractions.

Tobacco is a kind of high-biomass cash crop where the above-ground portions of the plant are harvested, and it has substantial soil nutrient demands. Specifically, in continuous tobacco cropping systems, tobacco plants selectively absorb certain soil nutrients, leading to reductions in soil mineral nitrogen (NH_4_^+^–N and NO_3_^−^–N), available potassium (AK), and SOC (Wang et al., 2022). Additionally, due to the high lignification of tobacco roots and the risk of soil-borne diseases, tobacco residues are not suitable for direct field return (Chen et al., 2018; Wang et al., 2017b). Consequently, long-term continuous cultivation often results in soil degradation, ultimately reducing crop yield and quality. Given this background, we hypothesize that granulated straw combined with an appropriate tillage method can serve as an effective strategy for improving soil fertility in tobacco fields. However, the effects of different incorporation amounts and burial depths on soil nutrients, SOC fractions, and microbial communities remain poorly understood. Here, two different tillage methods (deep tillage and rotary tillage) combined with three straw granulation amounts of low, medium, and high were set to explore 1) changes in soil available nutrients, SOC fractions, and soil bacterial community under different treatments; 2) the interactions between soil available nutrients, SOC fractions, and soil bacterial communities; and 3) an optimal granulated straw incorporation strategy to improve soil quality and enhance tobacco yield.

## 2 Materials and methods

### 2.1 Site description

A 3-year field experiment was conducted in Jiayue Town, Zhucheng City, Shandong Province, China (119°06′E, 36°01′N) from 2016 to 2018. The local altitude is 130 m. The climate is temperate monsoon, with an average annual temperature of 12.3°C, average annual sunshine hours of 2,578.4 h, and average annual precipitation of 773 mm. The annual frost-free period is 232 days. The cropping system is continuous cultivation of flue-cured tobacco, and the local tillage method is rotary tillage. The soil type is classified as leached cinnamon soil according to the Chinese Soil Taxonomy, with 12.51% sand, 44.65% silt, and 42.90% clay. The properties of soil initially at different layers are given in **Table 1**.

**Table 1:** Basic soil properties in different soil layers (Oct 2016).

Soil properties	Soil layer	
	0 - 20 cm	20 - 40 cm
Sand (>0.05 mm, %)	12.51 ± 0.21	12.57 ± 0.25
Slit (0.05–0.002 mm, %)	44.65 ± 2.52	42.42 ± 2.20
Clay (<0.002 mm, %)	42.90 ± 1.28	45.11 ± 1.67
Sand (>0.05 mm, %)	12.51 ± 0.35	12.57 ± 0.41
SOC (g kg^−1^)	8.58 ± 0.14	7.95 ± 0.10
AN (mg kg^−1^)	91.00 ± 2.97	66.00 ± 2.03
AP (mg kg^−1^)	20.06 ± 0.77	10.62 ± 0.54
AK (mg kg^−1^)	213.59 ± 3.31	208.35 ± 3.09
pH	7.89 ± 0.11	8.03 ± 0.14

Values represent mean ± SE (standard error, n = 3).

SOC, soil organic carbon; AN, available nitrogen; AP, available phosphorus; AK, available potassium.

### 2.2 Experimental design

Three kinds of granulated straw amounts (G1, 2,250; G2, 4,500; and G3, 6,750 kg hm^−2^) combined with two tillage modes (T, alternate-year deep tillage to a depth of 35 cm; and R, annual rotary tillage to a depth of 15 cm) were established. The conventional rotary tillage treatment without straw addition was set as the control (RG0). The experimental design diagram is shown in **Supplementary Figure S1**. Each treatment plot was 72 m^2^ (6 m × 12 m). All plots were arranged in a randomized complete block design with three replications. The granulated straw was prepared following the method of Wang et al. (2017a). The maize straw used for granulation contained 42.69% of carbon, 1.15% of nitrogen, 0.10% of phosphorus, and 0.98% of potassium. Additionally, chemical fertilizers and decomposition agents were added to the straw at a rate of 5.5 kg of NH_4_^+^–N, 2.0 kg of NO_3_^−^–N, 7.5 kg of P_2_O_5_, 6.0 kg of K_2_O, and 3.0 kg of decomposition agents per ton of straw. Since continuous deep tillage may damage soil structure, it was conducted every 2 years (specifically in 2016 and 2018). In the TG (deep tillage with granulated straw) treatment group, granulated straw was incorporated into the soil using a straw-burying plough with a 73.5 kW traction tractor (Model 1004) to a depth of approximately 35 cm. As the straw incorporation was carried out simultaneously with deep tillage, it was also applied biennially to align with the deep tillage schedule. In the RG (rotary tillage with granulated straw) treatment, straw was mixed with the topsoil (0–15 cm) using rotary tillage in 2016 and 2018. After that, ridging and strip fertilization were performed. The total amount of chemical fertilizer application was 76.95 kg hm^−2^ of N, 76.95 kg hm^−2^ of P_2_O_5_, and 193.50 kg hm^−2^ of K_2_O, which included the chemical fertilizer added during the preparation of granulated straw. The experimental flue-cured tobacco was NC55, which was transplanted in the first 10 days of May every year. The tobacco row and plant spacings were set at 120 and 50 cm, respectively, resulting in a total of 120 plants per plot.

### 2.3 Soil sampling and measurement

In 2016, 2017, and 2018, five soil samples were collected from each plot at depths of 0–20 and 20–40 cm. These five samples from each plot were combined to create a composite sample, which was then sieved through a 2-mm mesh to remove roots and plant debris (Ren et al., 2016). For available nutrient determination, samples were collected on the 20th, 40th, 60th, 80th, and 100th days after tobacco transplanting. Samples were collected after tobacco harvest and immediately stored at 4°C for the analysis of SOC, dissolved organic carbon (DOC), microbial biomass carbon (MBC), and available nutrients. For microbial analysis, soil samples were collected in September 2018 from the 20–40-cm depth and stored at −80°C for microbial DNA extraction.

#### 2.3.1 Determination of SOC fractions

The SOC content was determined using the K_2_Cr_2_O_7_–FeSO_4_ oxidation method with heating in an oil bath (Lu, 2000). Soil MBC content was measured using the chloroform–fumigation extraction technique (Vance et al., 1987). Meanwhile, DOC content was determined using the non-fumigated samples during the MBC extraction process (Dong et al., 2009). Both DOC and MBC concentrations were analyzed using a TOC analyzer (multi N/C 3100, Analytik Jena AG, Jena, Germany).

#### 2.3.2 Determination of soil available nutrients

AK was extracted using ammonium acetate and quantified by flame photometry. The NH^+^–N and NO_3_^−^–N were extracted with KCl solution (1 mol L^−1^), and the filtrates were analyzed using a continuous flow analyzer (AA3, SEAL Analytical, Norderstedt, Germany) according to Bao (2020).

#### 2.3.3 DNA extraction, sequencing, and bioinformatics analyses

Bacterial DNA was extracted from 0.5 g of fresh soil using the Fast^®^ DNA SPIN Kit (MP Biomedicals, Santa Ana, CA, USA). The V3–V4 region of the 16S rRNA gene was amplified with bacterial primers 338F (5′-ACTCCTAGGGAGGAGCA-3′) and 806R (5′-GGACTCHVGGGTWTTAT-3′). The PCR products were purified with the Agarose Gel DNA Purification Kit (TaKaRa, Shanghai, China). High-through paired-end sequencing of the DNA samples was performed at Majorbio Bio Technology Co., Ltd. (Shanghai, China). The raw sequences were analyzed using the QIIME software (v 1.9.0). After quality filtering, reads were clustered into operational taxonomic units (OTUs) using the closed-reference OTU picking against the Greengene database in QIIME (gg_13_5 version). Then, the community composition of each sample was assessed at each level. Mothur was used to analyze the α diversity of the bacterial communities (Chao1, Simpson, and Shannon indices).

### 2.4 Determination of flue-cured tobacco yield

The yield of flue-cured tobacco was determined by assessing the productive leaves from 50 plants located at the center of each plot. Tobacco leaves were harvested based on their maturity, and any unqualified leaves were discarded after inspection. The remaining leaves were then flue-cured and weighed, with the final measured mass representing the tobacco yield (Hu W. et al., 2021; Li et al., 2025).

### 2.5 Statistical analysis

SOC fractions and soil available nutrients were analyzed using one-way and two-way ANOVA in SAS 9.4. One-way ANOVA was used to compare differences among seven treatments, with significance determined by Least Significant Difference (LSD) at *p* < 0.05. Two-way ANOVA was used to assess the interaction between tillage methods and straw particle amounts. Principal component analysis (PCA) and permutational multivariate analysis of variance (PERMANOVA) were performed using the “vegan” package in R (v 4.4.0) to examine bacterial community differences based on the Bray–Curtis dissimilarity. Pearson’s correlation, calculated using the “heatmap” package in R, was used to explore the relationships between tobacco yield and soil properties in 2018. In addition, the relationships were further determined using a linear regression model with the Origin 2024 software. Box plots were also created using Origin 2024.

Microbial co-occurrence networks were constructed using CoNet to calculate multiple correlations between taxa and soil factors (Zheng et al., 2022). A valid co-occurrence was deemed statistically robust when the correlation coefficient exceeded 0.50 and the *p*-value was below 0.05. The *p*-values were adjusted using the Benjamini–Hochberg procedure to minimize false positives (Benjamini and Hochberg, 1995). Networks were visualized in the Gephi software (Bastian et al., 2009), where nodes represent OTUs and soil factors and edges indicate pairwise correlations between nodes (Weiss et al., 2016).

Structural equation modeling (SEM) was performed using SPSS-AMOS to analyze hypothetical pathways while explaining the interaction among SOC fractions, soil available nutrients, and soil bacterial traits under different granulated straw incorporation treatments. In general, a good fit of the SEM model meets most of the evaluation indices, including a lower chi-square/df ratio than 2 or 5 (Schumacker and Lomax, 2004), a higher *p*-value of the t-test than 0.05 (Marsh and Hocevar, 1985), a higher goodness-of-fit index (GFI) than 0.90 (Kaveh et al., 2023), and a lower standardized root mean square residual (SRMR) than 0.08 (Hu and Bentler, 1998; Shen et al., 2023).

## 3 Results

### 3.1 SOC fractions

**Figure 1:**
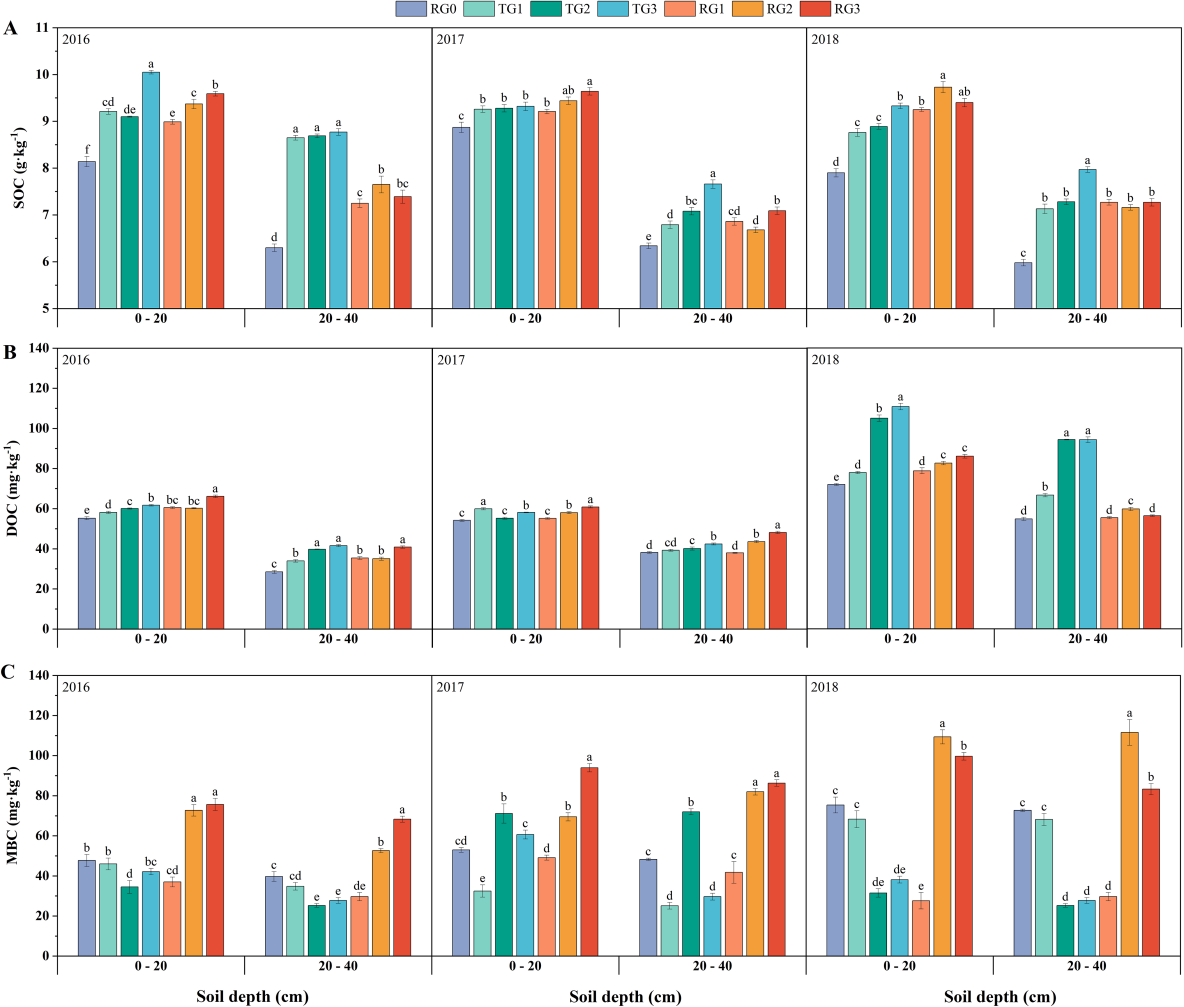
Changes in SOC **(A)**, DOC **(B)**, and MBC **(C)** in the 0–20- and 20–40-cm soil layers from 2016 to 2018. RG0, rotary tillage with no straw return; TG1, deep tillage with granulated straw 2,250 kg hm^−2^; TG2, deep tillage with granulated straw 4,500 kg hm^−2^; TG3, deep tillage with granulated straw 6,750 kg hm^−2^; RG1, rotary tillage with granulated straw 2,250 kg hm^−2^; RG2, rotary tillage with granulated straw 4,500 kg hm^−2^; RG3, rotary tillage with granulated straw 6,750 kg hm^−2^; SOC, soil organic carbon; DOC, dissolved organic carbon; MBC, microbial biomass carbon. Values represent mean ± SE (n = 3). Different lowercase letters indicate a significant difference between treatments at *p* < 0.05.

Granulated straw incorporation significantly increased SOC content (**Figure 1A**). In the 0–20-cm soil layer, increases were most pronounced under higher straw amount treatments (RG2 and RG3), with effects becoming more prominent over time. In the 20–40-cm soil layer, SOC content was the highest under the TG treatments and increased with increasing straw amount. Two-way ANOVA showed that both tillage methods (TM) and straw amounts (SA) had significant effects on SOC content (*p* < 0.01) in the 0–20-cm layer over all 3 years (**Supplementary Table S1**), while TM was the primary influencing factor in the 20–40-cm layer.

Compared with RG0, the RG3 treatment significantly increased DOC content in both the 0–20- and 20–40-cm soil layers in 2016 and 2017 (**Figure 1B**). In 2018, TG3 had the most significant increase in DOC content in both soil layers. Two-way ANOVA revealed that in the 0–20-cm soil layer, SA and the interaction of TM × SA significantly affected DOC content (*p* < 0.01) across all 3 years (**Supplementary Table S2**). In the 20–40-cm soil layer, DOC was significantly influenced by SA, TM, and their interaction (*p* < 0.05).

The DOC/SOC is an important indicator reflecting the impact of different soil managements on organic matter, which can predict the long-term change of soil organic matter (Ghani et al., 2003). RG3 had the highest DOC/SOC in 2016 and 2017, while TG2 and TG3 had the highest values in 2018 (**Supplementary Figure S2**).

Compared with RG0, the treatments of RG2 and RG3 significantly enhanced soil MBC content in both soil layers over the 3 years (**Figure 1C**). In addition, the two-way ANOVA showed that the TM, SA, and their interaction had significant effects on MBC content (*p* < 0.01) over the 3 years (**Supplementary Table S3**). The impact of each treatment on MBC/SOC is similar to that on MBC (**Supplementary Figure S2**).

### 3.2 Soil available nutrients

**Figure 2:**
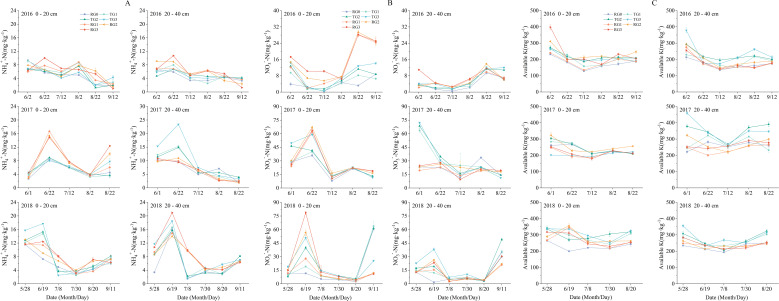
Changes in NH_4_^+^–N content **(A)**, NO_3_^−^–N content **(B)**, and available K content **(C)** in the 0–20- and 20–40-cm soil layers from 2016 to 2018. Values represent mean ± SE (n = 3). See **Figure 1** for detailed treatment notes.

During the growth period of flue-cured tobacco, the soil NH_4_^+^–N content gradually decreased but slightly increased at the maturity stage in both soil layers (**Figure 2A**). In 2016 and 2018, the RG2 and RG3 treatments had the highest average increases of NH_4_^+^–N in both soil layers. In 2017, the TG treatments had a higher average increase in NH_4_^+^–N than the RG treatments in the 20–40-cm soil layer (**Supplementary Figure S3**).

Granulated straw incorporation also promoted an increase in soil NO_3_^−^–N content (**Figure 2B**). RG3 showed the highest average increase level in both soil layers in 2016 (**Supplementary Figure S4**). In 2017, compared with the RG treatments, the TG treatments promoted the average increase in NO_3_^−^–N content during the growth period of flue-cured tobacco. In 2018, during the vigorous growing period (June 19), RG3 had the highest NO_3_^−^–N content in the 0–20-cm soil layer, while TG3 had the highest NO_3_^−^–N content in the 20–40-cm soil layer.

AK content exhibited an initial descending trend, followed by a subsequent ascending trend, during the growth period of flue-cured tobacco (**Figure 2C**). In the 20–40-cm soil layer, the TG3 treatment had the highest average increases of AK across all 3 years (**Supplementary Figure S5**).

### 3.3 Diversity, composition, and structure of bacterial community

Bacterial community analyses were conducted on the 20–40-cm soil layer due to substantial changes in nutrient and carbon components at this depth. A total of 1,830–2,121 OTUs were identified (**Table 2**), with RG2 exhibiting the highest richness, followed by RG3, while TG1 was the lowest (*p* < 0.05). The Chao1 and Shannon indices showed a similar trend, whereas the Simpson index showed the opposite pattern, with TG1 being the highest and RG2 the lowest (*p* < 0.05).

**Table 2:** The bacterial community diversity indices and the number of observed OTUs in different treatments.

Treatment	Richness index		Diversity index	
	OTUs	Chao1	Shannon	Simpson
RG0	1,912 ± 23.38c	2,501 ± 45.68c	5.853 ± 0.013cd	0.0121 ± 0.0002ab
TG1	1,830 ± 6.35d	2,473 ± 23.91c	5.731 ± 0.071d	0.0148 ± 0.0019a
TG2	1,910 ± 11.79c	2,617 ± 10.40b	5.863 ± 0.074cd	0.0118 ± 0.0025ab
TG3	1,955 ± 19.84c	2,622 ± 19.05b	5.919 ± 0.025bc	0.0111 ± 0.0008abc
RG1	1,945 ± 6.56c	2,615 ± 38.36b	5.899 ± 0.044cd	0.0135 ± 0.0006ab
RG2	2,121 ± 17.37a	2,719 ± 35.38a	6.267 ± 0.063a	0.0072 ± 0.0014c
RG3	2,045 ± 22.70b	2,736 ± 17.44a	6.078 ± 0.063b	0.0096 ± 0.0005bc

Values represent mean ± SE (n = 3). Different lowercase letters in the same column indicate a significant difference between treatments at *p* < 0.05.

RG0, rotary tillage with no straw return; TG1, deep tillage with granulated straw 2,250 kg hm^−2^; TG2, deep tillage with granulated straw 4,500 kg hm^−2^; TG3, deep tillage with granulated straw 6,750 kg hm^−2^; RG1, rotary tillage with granulated straw 2,250 kg hm^−2^; RG2, rotary tillage with granulated straw 4,500 kg hm^−2^; RG3, rotary tillage with granulated straw 6,750 kg hm^−2^; OTUs, operational taxonomic units.

**Figure 3:**
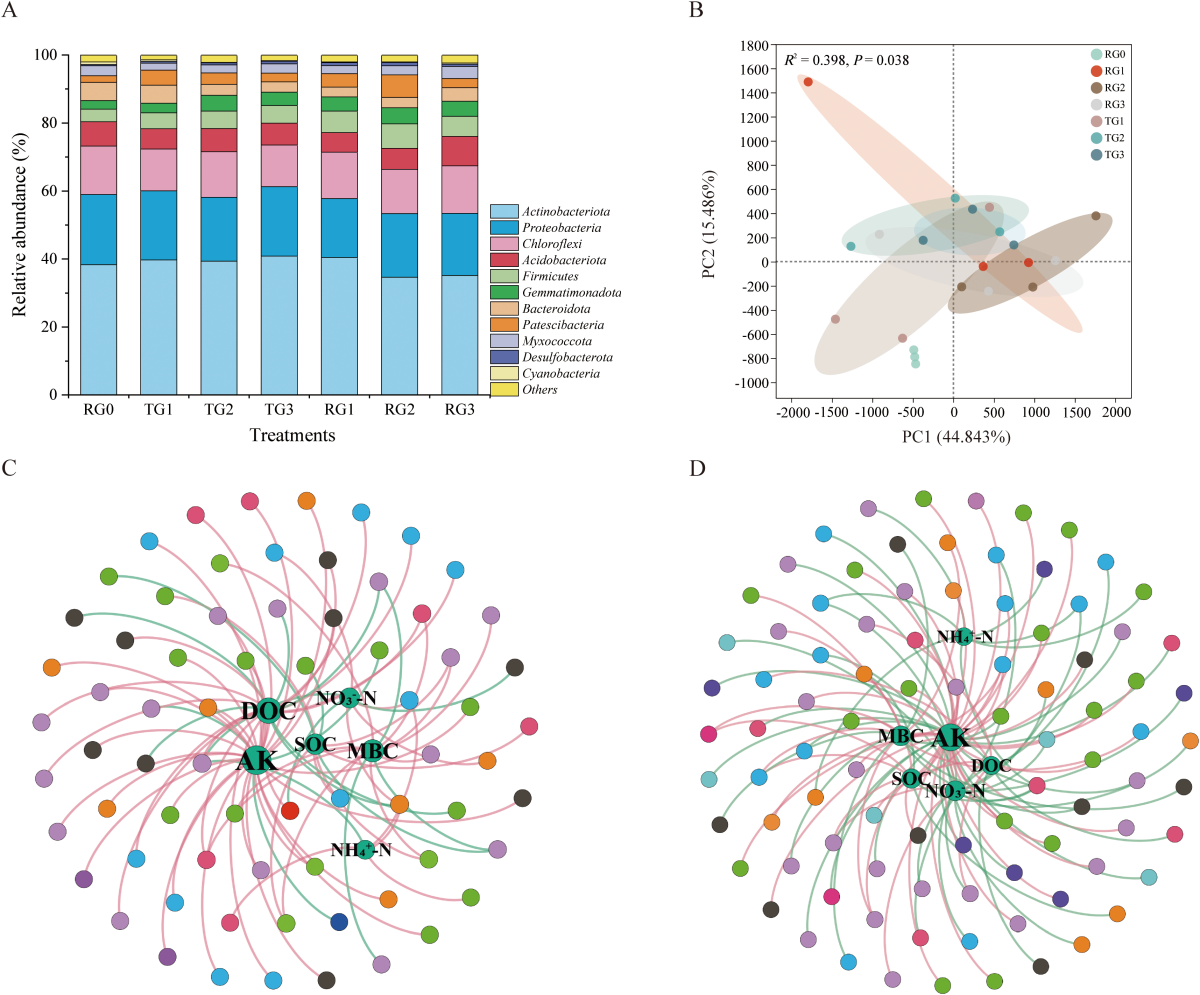
Dominant soil bacterial phyla under different treatments **(A)**. Principal component analysis of bacterial communities under different treatments **(B)**. Panel A presents the mean relative abundances (n = 3). In panel B, the x- and y-axes represent the first and second principal coordinates, respectively, and the values in parentheses show the percentage of the community variation explained. Network co-occurrence analysis of OTUs and environmental factors under deep tillage **(C)** and rotary tillage **(D)** treatments. Peripheral colored nodes represent OTUs assigned to major phyla. The environmental factor is located at the core and labeled. Positive correlations are shown as red edges, and negative correlations are shown as green edges. See **Figure 1** for detailed treatment notes; OTUs, operational taxonomic units.

The dominant bacterial phyla across treatments were Actinobacteriota (34.6%–40.8%), Proteobacteria (17.3%–20.6%), Chloroflexi (12.2%–14.2%), Acidobacteriota (5.7%–8.6%), Firmicutes (3.7%–7.2%), Gemmatimonadota (2.5%–3.8%), and Bacteroidota (2.8%–5.3%), accounting for 87.1%–91.7% of the total community (**Figure 3A**). Tillage methods had a stronger influence than straw amount on the relative abundances of Firmicutes, Spirochaetota, and Abditibacteriota (*p* < 0.05) (**Supplementary Figures S6A, B**). *NB1-j* had a significant difference among the TG treatments, while Spirochaetota had a significant difference among the RG treatments (**Supplementary Figures S6C, D**). The PCA explained 60.32% of the total variation, indicating distinct bacterial clustering by treatment (**Figure 3B**). The PERMANOVA confirmed that the community structures of bacteria were significantly affected by tillage and granulated straw (*p* < 0.05).

Network analysis revealed the associations between soil factors and bacterial phyla. Under the deep tillage management (**Figure 3C**), AK was the most influential factor, affecting bacterial OTUs with 28 positive and five negative edges among 69 nodes. It was followed by DOC with 15 positive edges and seven negative edges. Both AK and DOC were generally positively correlated with bacterial phyla. Under the rotary tillage management (**Figure 3D**), AK remained the dominant factor, with 26 positive and 20 negative edges among 90 nodes. MBC and NO_3_^−^–N were secondary factors, with 14 positive and seven negative edges for MBC and four positive and 18 negative edges for NO_3_^−^–N. Generally, AK and MBC had positive relationships with most bacterial phyla, while NO_3_^−^–N had a negative relationship with most bacterial phyla.

### 3.4 Tobacco yield

**Figure 4:**
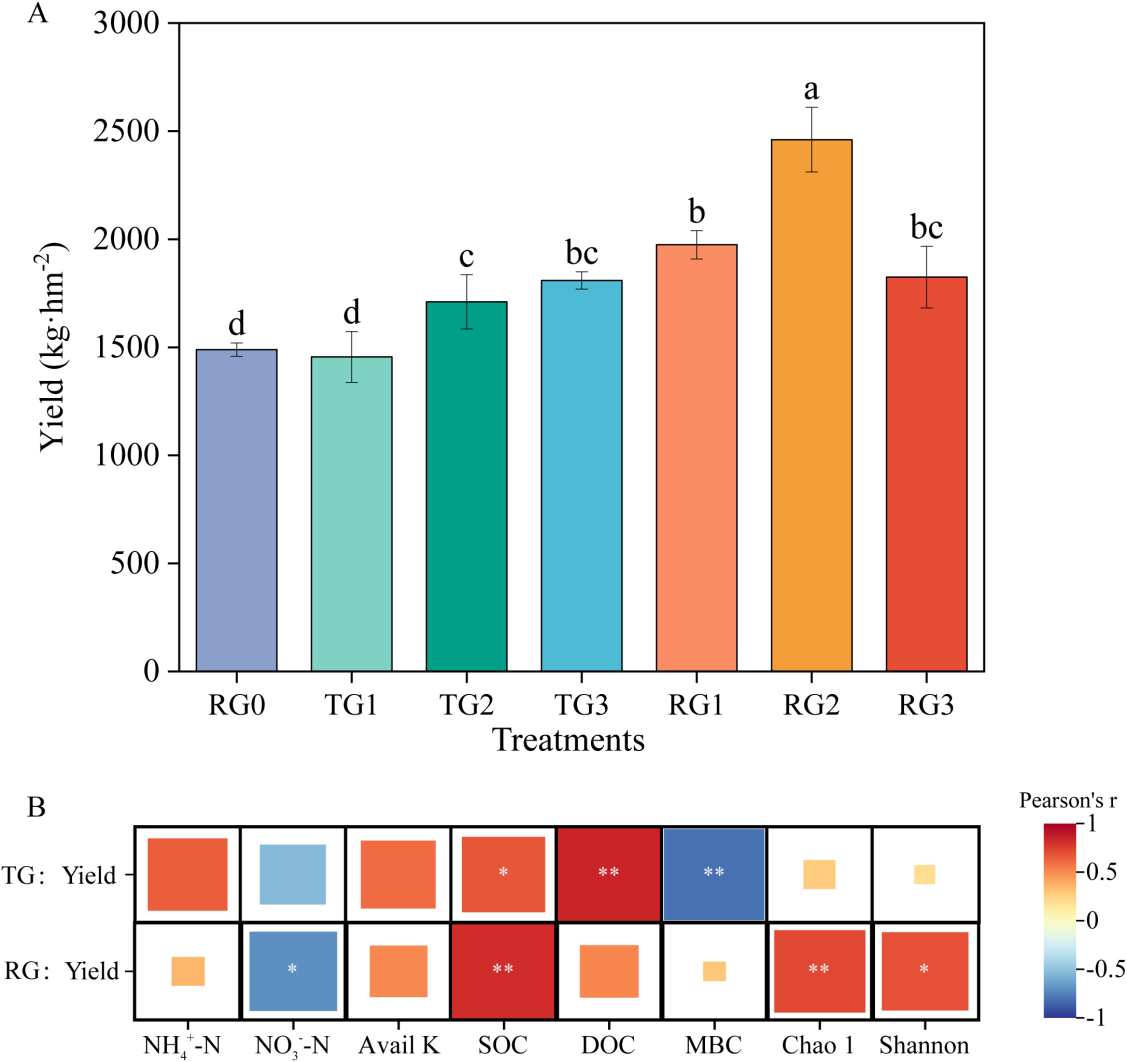
Tobacco yield under different tillage managements in 2018 **(A)**. Pairwise comparisons between tobacco yield and soil properties in 2018 **(B)**. Values represent mean ± SE (n = 3). Different lowercase letters indicate a significant difference between treatments at *p* < 0.05. The color saturation of each cell circle indicates Pearson’s rank correlation coefficients, and stars indicate the significance levels, with *p < 0.05 and **p < 0.01.

Compared with RG0, the RG treatments significantly improved tobacco yield, with RG2 exhibiting the highest increase of 65.1% (*p* < 0.05). Additionally, the high and medium straw amounts with deep tillage also resulted in significant yield improvements (**Figure 4A**). Pearson’s correlation analysis showed that the correlation between soil properties and yield varied significantly under different tillage methods (**Figure 4B**). Under deep tillage management, tobacco yield was most closely associated with SOC fractions. However, under rotary tillage management, yield was significantly correlated with NO_3_^−^–N, SOC, and bacterial Chao1 and Shannon indices. The linear regression analysis further confirmed that SOC and DOC were positively correlated with tobacco yield under deep tillage management (**Supplementary Figure S7A**). Moreover, significant positive correlations were also observed between yield and SOC, bacterial Chao1, and Shannon index (**Supplementary Figure S7B**).

### 3.5 The relationships among bacterial communities, soil available nutrients, and SOC fractions

**Figure 5:**
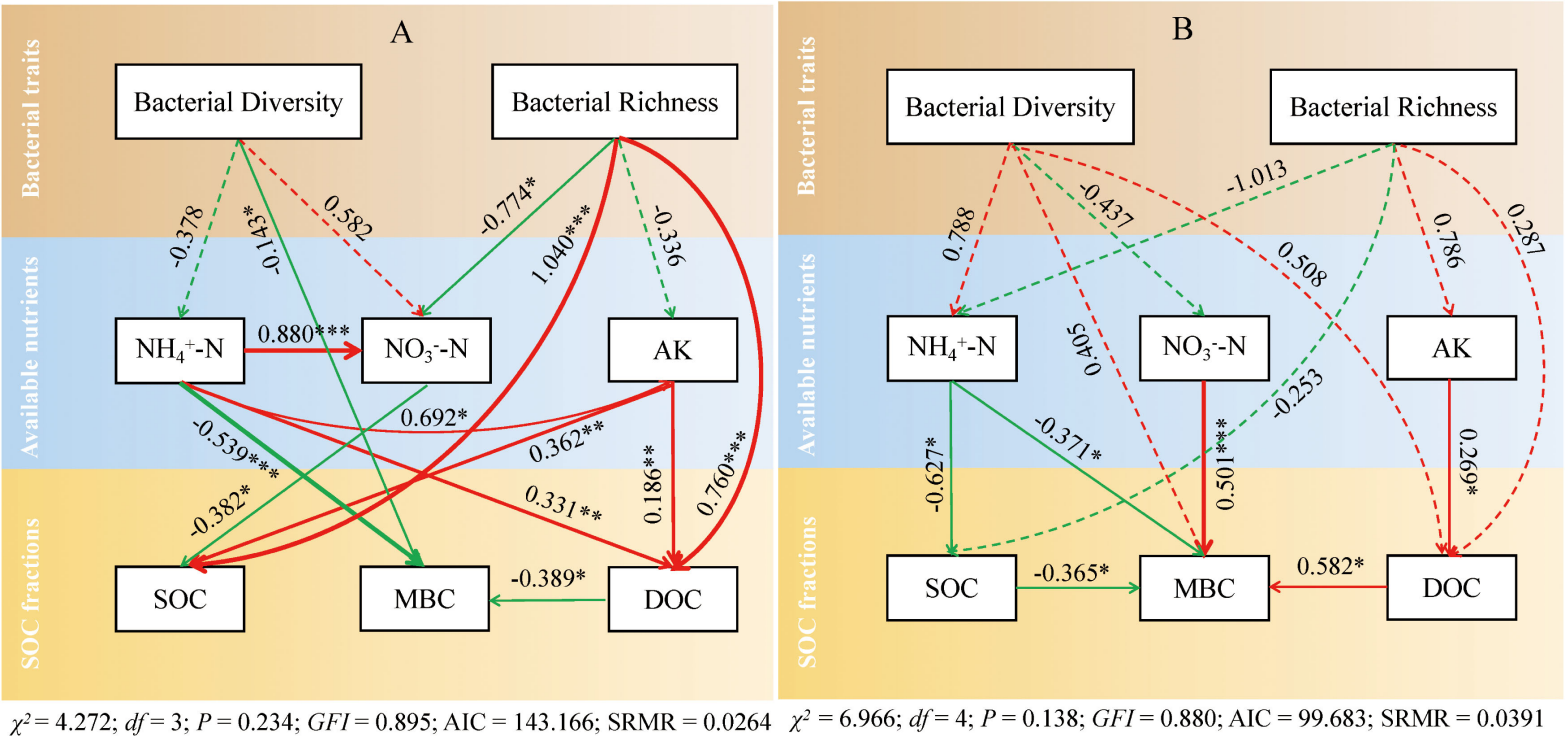
Structural equation modeling revealing the relationships among soil bacterial traits, available nutrients, and SOC fractions under deep tillage **(A)** and rotary tillage **(B)**. Square boxes denote variables included in the models. Values associated with solid arrows represent standardized path coefficients (SPCs), and asterisks mark their significance: *p < 0.05; **p < 0.01; ***p < 0.001. The arrow width is proportional to the strength of the path coefficients. Solid red arrows indicate a positive correlation, while solid green arrows indicate a negative relationship. The dotted arrows indicate the insignificant relationship with a high path coefficient. The data for the bacterial composition were calculated by principal component analysis. SOC, soil organic carbon.

SEM adequately fitted the data to assess the direct and indirect relationships among bacterial communities, soil available nutrients, and SOC fractions (**Figure 5**). Under the deep tillage management (**Figure 5A**), bacterial richness positively correlated with the SOC (Standardized Path Difference (SPC) = 1.040, *p* < 0.001) and DOC (SPC = 0.760, *p* < 0.001). Bacterial diversity directly affected MBC (SPC = −0.143, *p* < 0.001). In addition, NH_4_^+^–N directly affected DOC (SPC = 0.331, *p* < 0.01) and MBC (SPC = −0.539, *p* < 0.001). Under the rotary tillage management (**Figure 5B**), AK directly affected DOC (SPC = 0.269, *p* < 0.05), and NO_3_^−^–N was a main factor that affected MBC (SPC = 0.501, *p* < 0.001). NH_4_^+^–N negatively correlated with the SOC (SPC = −0.627, *p* < 0.05) and MBC (SPC = −0.371, *p* < 0.05).

## 4 Discussion

### 4.1 Effects of granulated straw incorporation with different tillage managements on SOC fractions

SOC is a key indicator of soil fertility and plays a crucial role in nutrient cycling (Bardgett et al., 2009). Numerous studies have confirmed that high straw input significantly increases SOC content (Cong et al., 2020; Liu N. et al., 2021). Similarly, our study demonstrated that incorporating granulated straw at medium and high amounts (4,500 and 6,750 kg hm^−2^, respectively) significantly enhanced SOC content. Beyond the straw amount, the tillage method was also a significant factor causing the difference in organic carbon. Many studies have shown that tillage will accelerate SOC mineralization, which is not conducive to improving the stability of the soil carbon pool (Zhang et al., 2019; Xu et al., 2019). In flue-cured tobacco systems, soil is frequently disturbed by cultivation and ridging prior to transplanting, which can further exacerbate carbon loss. Therefore, granulated straw was used to supplement the soil carbon pool in our study. The application of granulated straw in our study compensated for this loss, resulting in a net increase in SOC content, with the effect of straw incorporation outweighing the negative impact of tillage.

Soil DOC is an active component of SOC and a sensitive index reflecting soil fertility (Straathof et al., 2014). Since DOC is mainly derived from plant residues, straw input is a direct contributor to increased DOC content (Duval et al., 2018; Ren et al., 2023). Therefore, it is not surprising that straw return increased DOC content in our study. The proportion of DOC to SOC was generally high in 2018, especially in the TG2 and TG3 treatments, which was approximately 1.3%. This may be related to the reapplication of granulated straw in 2018. In particular, the TG treatment significantly increased the DOC content of the 20–40-cm soil layer, possibly due to enhanced aeration and permeability, which promote organic matter transformation and accumulation (Latifmanesh et al., 2020).

MBC is a sensitive indicator of changes in SOC pools due to agricultural practices and an available nutrient pool in soil (Kubar et al., 2019; Chen et al., 2017). In our study, higher straw incorporation with rotary tillage significantly elevated soil MBC content across 3 years. On the one hand, high input organic substrate was an important factor that increased MBC content (Yuan et al., 2021); on the other hand, the high incorporation amount with rotary tillage reduced the uneven distribution of granulated straw in the soil layer. Several studies have found that tillage practice led to a decrease in soil MBC (Giuseppe et al., 2015; Yang et al., 2015). This is because tillage increases soil aeration, which then promotes carbon mineralization, and decreases soil moisture, which then inhibits microbial growth (Yang et al., 2015). In particular, deep tillage had a strong disturbance of the soil, which could lead to serious microbial carbon loss.

### 4.2 Effects of granulated straw incorporation with different tillage managements on soil available nutrients

Our study demonstrated that the amount of granulated straw incorporation plays a crucial role in regulating mineral nitrogen availability in tobacco-planting soil. Specifically, medium and high incorporation amounts led to significant increases in available nitrogen. These results are consistent with previous studies reporting positive relationships between straw input and soil nutrient enrichment (Cong et al., 2019a; Cong et al., 2019b). Throughout the flue-cured tobacco growth period, soil NH_4_^+^–N content exhibited dynamic fluctuations, with an early peak accumulation occurring at the initial stage. This phenomenon may be attributed to humic acid formation during straw decomposition, which enhances the activation of fixed ammonium (Williams et al., 2018). Additionally, the relatively low nutrient demand of tobacco at early growth stages (Hu R. et al., 2021) may contribute to NH_4_^+^–N accumulation. In contrast, soil NO_3_^−^–N content declined in the middle growth period, likely due to leaching associated with rainfall. The subsequent peak in the later growth stage was most likely driven by enhanced nitrification, leading to an increase in NO_3_^−^–N content.

Potassium is a key determinant of tobacco leaf quality and is closely related to soil AK content. Our study found that AK content can be improved by different granulated straw incorporation methods. This improvement can be attributed to the relatively high potassium content (1%–2%) in maize straw and its efficient release due to the ionic nature of potassium in straw (Cong et al., 2020). Moreover, humic acids formed during the decomposition of granulated straw can activate fixed potassium. This can also explain why AK content increases with the increase in granulated straw amount. The TG treatments promoted AK accumulation more effectively than the RG treatments, likely because deep tillage facilitates nutrient transport to subsoil layers, breaks the plow pan, and enhances root penetration and exudate-driven nutrient mobilization (Giles et al., 2017).

### 4.3 Responses of bacterial traits to different granulated straw incorporation methods

The 20–40-cm soil layer was selected for microbial analysis due to its heightened sensitivity to carbon fractions and nutrient changes under different straw incorporation practices. Our sequencing results showed that bacterial richness was the highest under the RG1 and RG2 treatments. This suggests that higher carbon input and less disturbance to the 20–40-cm soil layer promoted the increase in bacterial richness (Muhammad et al., 2011; Bastida et al., 2006). Additionally, RG2 exhibited the highest Shannon index and the lowest Simpson index, indicating greater bacterial diversity and evenness under this treatment.

In our study, the granulated straw incorporation with different tillage methods for 3 years did not change the soil dominant bacterial phyla but had a significant impact on the less abundant phyla. Long-term tobacco cultivation may have contributed to a relatively stable microbial community structure, as plant root exudates play a crucial role in plant-to-plant signaling and strongly influence microbial community composition (Iannucci et al., 2021). Our study observed increased relative abundances of Firmicutes and Gemmatimonadota under rotary tillage. Firmicutes is the main microbial community that degrades straw under anaerobic conditions, and reduced disturbance in the 20–40-cm layer under the RG treatments may favor their proliferation (Qiao et al., 2013). Gemmatimonadota has the ability to perform aerobic methane oxidation and supports carbon fixation by encoding enzymes to use trace gases as electron donors (Bay et al., 2021). Methane gas generated under anaerobic conditions provides substrate for oxidation reaction participated by Gemmatimonadota, which may increase the relative abundance of Gemmatimonadota in the 20–40-cm soil layer. In the future study, we will further investigate the functional potential of these microbial communities through metagenomic analysis.

### 4.4 Effects of granulated straw incorporation with different tillage managements on tobacco yield

Straw incorporation is used to achieve higher economic output by improving crop yield (Song et al., 2024). Previous studies have demonstrated that a moderate rate of straw incorporation optimizes available nutrients and yields the highest crop production (Alijani et al., 2012). Therefore, it comes as no surprise that high and medium straw amounts significantly contributed to the increase in tobacco yield in our study. Indeed, organic amendments, such as straw, have been shown to enhance crop yield by improving soil microbial conditions (Liu J. A. et al., 2021). Our study supports this finding, as evidenced by the positive correlation between tobacco yield and both the bacterial Chao1 and Shannon indices. The RG2 treatment had the most significant effect on tobacco yield, likely due to the increased diversity of bacterial phyla involved in straw decomposition and carbon fixation. Overall, granulated straw incorporation with rotary tillage is an effective strategy to enhance tobacco yield by enhancing SOC content, bacterial richness, and diversity.

### 4.5 Relationships between SOC fractions and soil available nutrients with bacterial traits

Our study identified AK as the primary factor influencing bacterial community under different tillage practices, generally showing a positive correlation with most bacterial phyla. Liu et al. (2017) reported that AK is a major driver of bacterial community variation, following only pH and available phosphorus (AP). Similarly, Pan et al. (2020) also highlighted AK as a major determinant of shifts in bacterial community structure. This is likely because certain bacteria participate in solubilizing and cycling soil potassium, thereby selecting for potassium-associated taxa (Miransari, 2013). The widespread use of potassium sulfate in tobacco fields may further amplify the impact of AK on bacterial communities. In addition to AK, DOC was another dominant factor under deep tillage, while NO_3_^−^–N played a crucial role under rotary tillage. Deep tillage facilitates the incorporation of granulated straw into the 20–40-cm soil layer, significantly increasing DOC content, which in turn enhances microbial abundance by providing a readily available carbon source (Straathof et al., 2014). In contrast, rotary tillage causes minimal soil disturbance at this depth, leading to NO_3_^−^–N accumulation due to leaching. Under anaerobic conditions, this accumulation promotes denitrification, ultimately resulting in a negative correlation between NO_3_^−^–N and most bacterial phyla.

The SEM further confirmed that bacterial traits significantly affect both the soil available nutrients and SOC fractions. Under deep tillage management, bacterial richness exhibited a strong positive correlation with SOC and DOC. It is well established that granulated straw incorporation enhances soil microbial populations, thereby facilitating the conversion of straw-derived carbon into stable soil organic carbon pools (Yang et al., 2021). Compared to deep tillage, bacterial traits under rotary tillage exhibited weaker effects on soil available nutrients and SOC fractions, likely due to the minimal disturbance of the 20–40-cm soil layer. A notable observation was the negative correlation between NH_4_^+^–N and both SOC and MBC. Since the SEM was built based on sampling at harvest time (September), at this time, the high soil temperature and moisture content at this stage likely enhanced microbial activity (Tan et al., 2018), accelerating nitrification. This process contributed to a decrease in NH_4_^+^–N. Conversely, NO_3_^−^–N showed a positive correlation with MBC, suggesting that enhanced nitrification increased NO_3_^−^–N levels. Furthermore, our study confirmed a significant positive correlation between AK and DOC under both tillage methods, likely due to the interaction between plant root exudates and soil nutrients. After tobacco topping, root systems release potassium into the soil, thereby increasing AK levels (Dai et al., 2009). Simultaneously, high temperature and humidity conditions promote DOC production, ultimately driving a synergistic increase in soil AK and DOC. In our study, the SEM analysis was conducted using data collected in 2018, the third year of the experiment. While this provides valuable insights into the relationships among soil properties and microbial communities under relatively stable conditions, the lack of multi-year microbial data limits our ability to assess interannual variability. Future studies incorporating multi-year data would be beneficial for more fully capturing the dynamic nature of these interactions and providing a more comprehensive understanding of the long-term effects of different tillage measures.

## 5 Conclusions

Over the 3-year field experiment, the medium (4,500 kg hm^−2^) and high (6,750 kg hm^−2^) amounts of granulated straw incorporation improved the content of SOC fractions and available nutrients in both the 0–20- and 20–40-cm soil layers. Furthermore, the medium straw incorporation amounts with rotary tillage significantly increased the bacterial α diversity and the relative abundance of phyla Firmicutes and Gemmatimonadota in the 20–40-cm soil layer. Soil AK was the most important nutrient factor affecting different bacterial phyla. SEM revealed that SOC fractions under rotary tillage were primarily regulated by nutrient factors, while bacterial richness and AK played dominant roles under deep tillage. Medium granulated straw incorporation with rotary tillage is more worthy of improving the tobacco yield. These findings provide valuable insights for optimizing tillage practices to enhance soil quality and crop yield in tobacco cultivation systems.

## Data Availability

The datasets presented in this study are publicly available in the Figshare repository at the following DOI: https://doi.org/10.6084/m9.figshare.29567522.
