# Supplementary material for: Granulated straw incorporation with rotary tillage increases the content of soil organic carbon fractions and available nutrients and shifts bacterial communities in East China
Source: Front Plant Sci. 2025 Jul 31;16:1520760. doi: 10.3389/fpls.2025.1520760 (PMC12350379; doi:10.3389/fpls.2025.1520760)
Supplement: Supplementary file 1 [file DataSheet1.docx]

***Supplementary Material***

**Table S1** Effect of the interaction between tillage management and granulated straw with two-way ANOVA analysis on soil organic carbon

| Soil layers  (cm) | Year | TM | |  | GA | |  | TM × GA | |
| --- | --- | --- | --- | --- | --- | --- | --- | --- | --- |
|  |  | *F* | *P* |  | *F* | *P* |  | *F* | *P* |
| 0 - 20 | 2016 | 12.00 | 0.0061 |  | 130.55 | <0.0001 |  | 29.99 | <0.0001 |
|  | 2017 | 5.68 | 0.0384 |  | 5.14 | 0.0292 |  | 3.14 | 0.0875 |
|  | 2018 | 45.85 | <0.0001 |  | 10.66 | 0.0033 |  | 10.44 | 0.0036 |
| 20 - 40 | 2016 | 190.24 | <0.0001 |  | 1.84 | 0.2085 |  | 1.59 | 0.2519 |
|  | 2017 | 18.71 | 0.0015 |  | 25.98 | 0.0001 |  | 7.50 | 0.0103 |
|  | 2018 | 42.16 | <0.0001 |  | 52.17 | <0.0001 |  | 45.63 | <0.0001 |

Note: TM, tillage management; GA, granulated straw amount. TM×GA, the interaction between tillage management and granulated straw amount.

**Table S2** Effect of the interaction between tillage management and granulated straw with two-way ANOVA analysis on soil dissolved organic carbon

| Year | Soil layer  (cm) | TM | |  | GA | |  | TM × GA | |
| --- | --- | --- | --- | --- | --- | --- | --- | --- | --- |
|  |  | *F* | *P* |  | *F* | *P* |  | *F* | *P* |
| 0 - 20 | 2016 | 42.53 | <0.0001 |  | 62.77 | <0.0001 |  | 11.94 | 0.0022 |
|  | 2017 | 0.35 | 0.5699 |  | 16.97 | 0.0006 |  | 37.04 | <0.0001 |
|  | 2018 | 188.92 | <0.0001 |  | 177.30 | <0.0001 |  | 52.98 | <0.0001 |
| 20 - 40 | 2016 | 6.62 | 0.0278 |  | 53.36 | <0.0001 |  | 12.21 | 0.0021 |
|  | 2017 | 35.36 | <0.0001 |  | 78.47 | <0.0001 |  | 22.01 | 0.0002 |
|  | 2018 | 1800.68 | <0.0001 |  | 238.53 | <0.0001 |  | 162.77 | <0.0001 |

Note: TM, tillage management; GA, granulated straw amount. TM×GA, the interaction between tillage management and granulated straw amount.

**Table S3** Effect of the interaction between tillage management and granulated straw with two-way ANOVA analysis on soil microbial biomass carbon

| Year | Soil layer  (cm) | TM | |  | GA | |  | TM × GA | |
| --- | --- | --- | --- | --- | --- | --- | --- | --- | --- |
|  |  | *F* | *P* |  | *F* | *P* |  | *F* | *P* |
| 0 - 20 | 2016 | 188.18 | <0.0001 |  | 45.81 | <0.0001 |  | 97.02 | <0.0001 |
|  | 2017 | 45.37 | <0.0001 |  | 87.52 | <0.0001 |  | 17.85 | 0.0005 |
|  | 2018 | 188.05 | <0.0001 |  | 36.45 | <0.0001 |  | 238.80 | <0.0001 |
| 20 - 40 | 2016 | 276.98 | <0.0001 |  | 52.99 | <0.0001 |  | 117.01 | <0.0001 |
|  | 2017 | 168.85 | <0.0001 |  | 138.97 | <0.0001 |  | 46.47 | <0.0001 |
|  | 2018 | 142.33 | <0.0001 |  | 15.81 | 0.0008 |  | 169.03 | <0.0001 |

Note: TM, tillage management; GA, granulated straw amount. TM×GA, the interaction between tillage management and granulated straw amount.

**Figure S1**

**
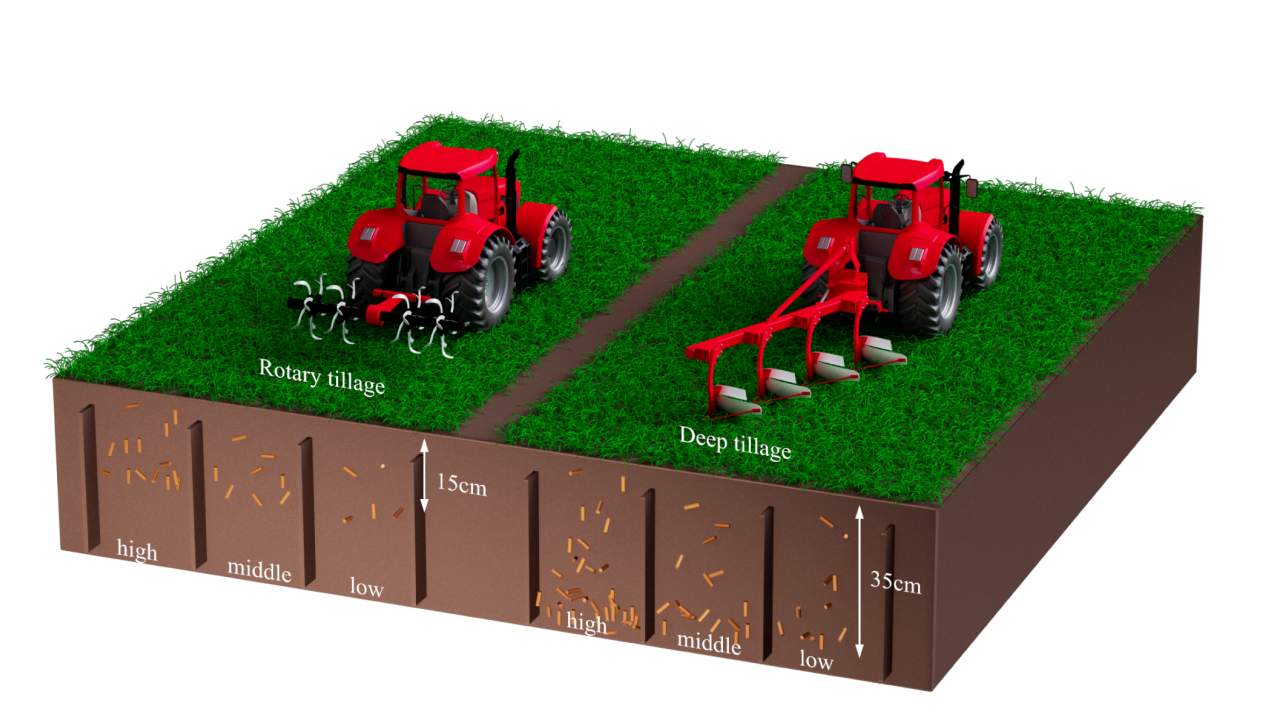
**

**Supplementary Figure 1.** Experimental design diagram. The experiment designed three levels of granulated straw incorporation rates: low, medium, and high, which were 2250 kg hm^-2^, 4500 kg hm^-2^, and 6750kg hm^-2^, respectively. In the Deep tillage treatment group, granulated straw was incorporated into the soil using a straw-burying plough with a 73.5 kW traction tractor (Model 1004) to a depth of approximately 35 cm. In the Rotary tillage treatment, straw was mixed with the topsoil (0 – 15 cm) using rotary tillage.

**Figure S2**

**Supplementary Figure 2.** Changes of DOC/SOC(A) and MBC/SOC(B) in 0 - 20 cm and 20 - 40 cm soil layers from 2016 to 2018. RG0, rotary tillage with no straw return; TG1, deep tillage with granulated straw 2250 kg hm^-2^; TG2, deep tillage with granulated straw 4500 kg hm^-2^; TG3, deep tillage with granulated straw 6750 kg hm^-2^; RG1, rotary tillage with granulated straw 2250 kg hm^-2^; RG2, rotary tillage with granulated straw 4500 kg hm^-2^; RG3, rotary tillage with granulated straw 6750 kg hm^-2^. SOC, soil organic carbon; DOC, dissolved organic carbon; MBC, microbial biomass carbon. Values represent mean ± SE (n = 3).

**Figure S3**

|  |  |
| --- | --- |

**Supplementary Figure 3.** Average increase of NH_4_^+^-N content during the growing period flue-cured tobacco. RG0, rotary tillage with no straw return; TG1, deep tillage with granulated straw 2250 kg hm^-2^; TG2, deep tillage with granulated straw 4500 kg hm^-2^; TG3, deep tillage with granulated straw 6750 kg hm^-2^; RG1, rotary tillage with granulated straw 2250 kg hm^-2^; RG2, rotary tillage with granulated straw 4500 kg hm^-2^; RG3, rotary tillage with granulated straw 6750 kg hm^-2^. Values represent mean ± SE (n = 3).

**Figure S4**

|  |  |
| --- | --- |

**Supplementary Figure 4.** Average rate of NO_3_^-^-N content during the growing period flue-cured tobacco. RG0, rotary tillage with no straw return; TG1, deep tillage with granulated straw 2250 kg hm^-2^; TG2, deep tillage with granulated straw 4500 kg hm^-2^; TG3, deep tillage with granulated straw 6750 kg hm^-2^; RG1, rotary tillage with granulated straw 2250 kg hm^-2^; RG2, rotary tillage with granulated straw 4500 kg hm^-2^; RG3, rotary tillage with granulated straw 6750 kg hm^-2^. Values represent mean ± SE (n = 3).

**Figure S5**

|  |  |
| --- | --- |

**Supplementary Figure 5.** Average rate of available K content during the growing period flue-cured tobacco. RG0, rotary tillage with no straw return; TG1, deep tillage with granulated straw 2250 kg hm^-2^; TG2, deep tillage with granulated straw 4500 kg hm^-2^; TG3, deep tillage with granulated straw 6750 kg hm^-2^; RG1, rotary tillage with granulated straw 2250 kg hm^-2^; RG2, rotary tillage with granulated straw 4500 kg hm^-2^; RG3, rotary tillage with granulated straw 6750 kg hm^-2^. Values represent mean ± SE (n = 3).

**Figure S6**


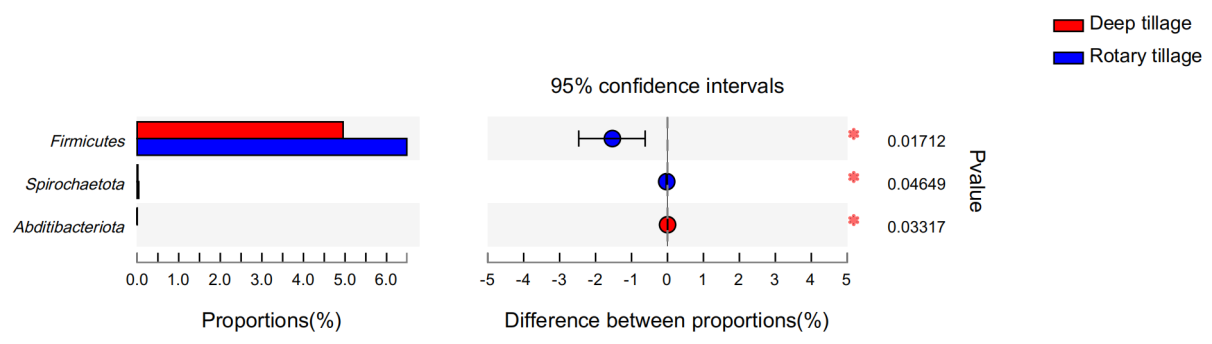


A

B


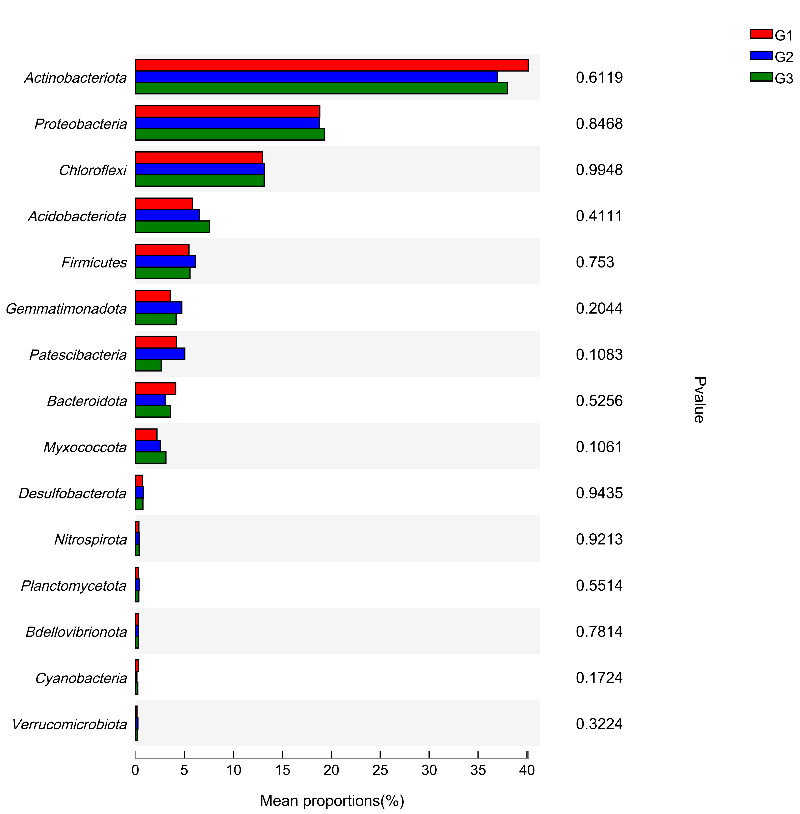


C


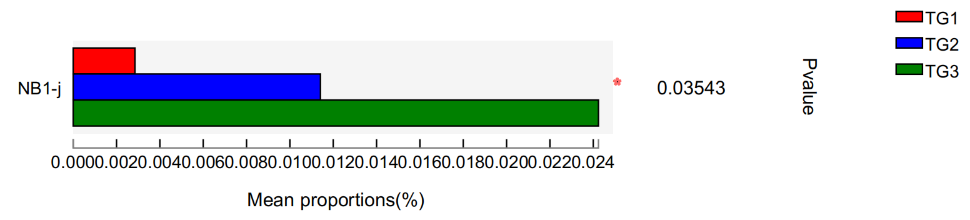


D


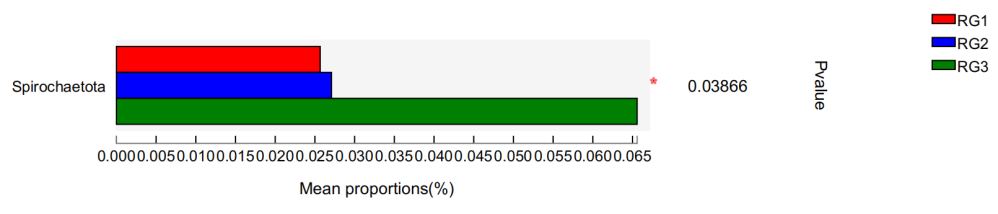


**Supplementary Figure 6.** Inter group differences of bacterial abundance among different tillage methods (A, n = 9, * *P* < 0.05) and granulated straw amounts (B, n = 6) at the phylum level based on the significance test analysis of Welch’s tests, the data are expressed as mean values. C and D are the significance test analysis based on one-way ANOVA between deep tillage and rotary tillage under three granulated straw amounts. The data are expressed as the average value, n = 3, * *P* < 0.05. RG0, rotary tillage with no straw return; TG1, deep tillage with granulated straw 2250 kg hm^-2^; TG2, deep tillage with granulated straw 4500 kg hm^-2^; TG3, deep tillage with granulated straw 6750 kg hm^-2^; RG1, rotary tillage with granulated straw 2250 kg hm^-2^; RG2, rotary tillage with granulated straw 4500 kg hm^-2^; RG3, rotary tillage with granulated straw 6750 kg hm^-2^.

**Figure S7**

**Supplementary Figure 7.** Relationships between between tobacco yield in 2018 and soil properties under deep tillage (A) and rotary tillage (B). The solid and shadow indicated the linear regression lines and 95 % confidence intervals. Stars indicate the significance levels of LSD, with: **P* < 0.05; ***P* < 0.01.
